# Breast cancer occurrence after low dose radiotherapy of non-malignant disorders of the shoulder

**DOI:** 10.1038/s41598-019-41725-w

**Published:** 2019-03-28

**Authors:** Felix Zwicker, Corinna Kirchner, Peter E. Huber, Jürgen Debus, Hansjörg Zwicker, Rudolf Klepper

**Affiliations:** 1 Clinical Cooperation Unit Molecular Radiation Oncology, German Cancer Research Center (DKFZ), Heidelberg, Germany; 2 Clinic and Practice of Radiation Oncology/Practice of Radiology, Konstanz, Germany; 3 Department of Radiation Oncology, University of Heidelberg, Heidelberg, Germany

## Abstract

Stochastic long-term damages at relatively low doses have the potential for cancer induction. For the first time we investigated the occurrence of breast cancer in female patients after radiotherapy of non-malignant disorders of the shoulder and made a comparison with the estimated spontaneous incidence of mammary carcinoma for this cohort. In a geographically defined district with a population of approximately 100.000 inhabitants, comprehensive data of radiological diagnostics and radiotherapy were registered nearly completely for 41 years; data included mammography and radiotherapy of breast cancer patients as well as of non-malignant disorders. Within this population a collective of 158 women with radiotherapy of the shoulder was investigated. Radiotherapy was performed with cobalt-60 photons (Gammatron) with an average cumulative-dose of 6 Gy. The average follow-up time was 21.3 years. Patients were 55 years old (median) when radiotherapy of the shoulder was performed. Seven patients (4.4%) developed breast cancer after a median of 21 years. According to the incidence statistics, 9.4 +/− 1.8 (95%CI) cases (5.9%) would be expected. In regard to the irradiated shoulder neither the ipsilateral nor the contralateral breasts showed increased rates of breast cancer. An induction of additional breast cancer caused by radiation of non-malignant disorders of the shoulder wasn’t detected in the investigated cohort.

## Introduction

Potential cancer induction after low dose radiotherapy of non-malignant disorders is in clinical focus as well as in patients’ interest. This long-term study of breast cancer occurrence after low dose radiotherapy of non-malignant disorders of the shoulder will attempt to make a contribution to this thematic.

In addition to the common use of radiotherapy to treat malignant tumors low dose irradiation is used successfully for treatment of non-malignant disorders such as activated and symptomatic arthrosis or degenerative arthropathy^1,2^. In Germany about 30,000 patients are treated a year. Omarthrosis and the impingement syndrome are the most frequent indications for low dose radiotherapy of the shoulder with long-lasting response rates up to 70–80% leading to prolonged analgesia^3^. Common fractionation regimes use single doses of 0.5–1.0 Gy one or two times a week to cumulative doses of 3–6 Gy. In case of particular pain reduction, a second treatment series is often performed 6–12 weeks after the end of the first series, leading to decelerated response. Basically, the biological effect mechanism is based on cellular anti-inflammatory reactions^4–6^. It was possible to demonstrate that the adhesion ability between leucocytes and endothelial cells is reduced after low dose irradiation in a range of 0.3–1 Gy^7,8^. In conjunction with these findings, the protein TGF-beta-1, an inhibitor of endothelial cell adhesion, is up regulated^9,10^. After low dose irradiation also the induced nitric oxide synthethase (iNOS) and analogous the pro inflammatory nitric oxide (NO) were reduced in macrophages^11^. As well, apoptosis rates of inflammatory cells show their maximum peak between 0.5 and 1.0 Gy^12,13^ and correspond with the single doses used in clinical daily routine and in randomized clinical trials to treat non-malignant disorders with low dose radiotherapy^14^.

Soon after the discovery of ionizing radiation, detrimental effects on body cells and tissues became known. However, not only deterministic damages at a high dose, but also many stochastic long-term damages at relatively low doses have been observed and published^15–17^. An overview of the literature on induction of mammary carcinomas can be found in the publication of Nekolla^18^.

In spite of the variation in exposure conditions, such as whole body or body part irradiation or the great differences in dose and dose rate values, a simple mathematical algorithm to estimate the secondary malignancy risk has been constructed using the “effective dose” model^19^. Critics do not always consider this procedure to be effective and call for more epidemiological studies to record disease risks, in particular in non-malignant radiation therapy treatments with relatively low doses^4,20^. Old patient collectives with middle dose radiotherapy (15–35 Gy) of benign gynecologic or gastric disorders showed increased rates of hematologic and epithelial malignancies^21–24^.

In case of low dose radiotherapy of non-malignant disorders it is reported that treatment of ankylosing spondylitis by irradiation of the whole bone spine or in parts increases the risk of leukaemia and cancer. Applied cumulative doses ranged from 4 to 14 Gy leading to a mean total body dose of 2.6 Gy^25–27^.

In a Swedish cohort study the risk of haematological malignancies was investigated in patients treated with X-rays (3–9 Gy) for benign lesions in the locomotor system^28,29^. The mean absorbed red bone narrow dose was estimated for treatments of different parts of the skeleton and was correlated with the risk of haematological malignancies. In case of mean absorbed red bone narrow dose >0.2 Gy patients showed increased rates of haematological malignancies, particularly after treatment of ankylosing spondylitis. On the other hand low dose irradiation of the shoulder caused a mean absorbed red bone narrow dose of 0.11 Gy and did not raise rates of haematological malignancies. Other peripheral articulations showed much more lower mean absorbed red bone narrow dose and as well no increased rates of haematological malignancies^28,29^.

In newer literature it is reported, that the carcinogenic risk of radiotherapy of benign diseases is decreased when treated patients are older than 40 years. The suggested life time risk of cancer induction is approximately 0.2% with low dose joint irradiation. The risk of cancer induction decreases further with increasing age of the patients^1,6,30,31^.

Clinical long term data with information about cancer induction after low dose radiotherapy of non-malignant disorders of peripheral articulations are very rare. To our knowledge clinical data about induction of breast cancer after radiotherapy of non-malignant disorders of the shoulder haven’t been published yet.

In a geographically defined district with a population of approximately 100.000 inhabitants, comprehensive data of radiological diagnostics and radiotherapy were registered nearly completely for 41 years; our radiological institute has provided a comprehensive range of radiological diagnostics including preventive mammography/screening mammography and of radiotherapy including breast cancer therapy and radiotherapy of non-malignant disorders.

While the data is not a formally prospective and controlled study, it constitutes a register study for approximately 100.000 people. Thus it is a completely observed longitudinal observation study. This work will attempt to make a contribution to this purpose. In this long-term analysis we investigated the occurrence of breast cancer in female patients after radiotherapy of non-malignant disorders of the shoulder and made a comparison with the estimated spontaneous incidence of mammary carcinoma for this cohort.

## Patients and Methods

### The cohort

In a geographically defined district with a population of approximately 100.000 inhabitants, comprehensive data of radiological diagnostics and radiotherapy were registered nearly completely for 41years. In this single-center study we retrospectively investigated all 158 women who were irradiated because of symptomatic arthrosis or degenerative arthropathy of the shoulder such as periarthritis humeroscapularis (PHS) in our institute in the period from 1976 to 1995 in Konstanz. The study has been carried out in accordance with The Code of Ethics of the World Medical Association (Declaration of Helsinki). The study was proofed by our institutional review board of the Clinic of Radiation Oncology in Konstanz.

According to the local ethics committee (Ethik-Kommission der Landesärztekammer Baden-Württemberg, Germany) formal consent is not required for this type of study (retrospective single centre analysis).

All patients were local residents of the district of Konstanz. Twenty-eight women who were irradiated on both shoulders were counted only once, with the earlier treatment.

For 41 years our institute in Konstanz has provided a comprehensive range of radiological diagnostics including preventive mammography/screening mammography and of radiotherapy including breast cancer therapy and radiotherapy of non-malignant disorders. In this function it is part of the Clinic of Konstanz and its Breast Cancer Center with a supra-regional service area. In our in-house archive patient data have been centralized since 1976. Of course, it was not a formally prospective and controlled study, but in fact the study was retrospectively performed similar to such a trail. So it is a completely observed longitudinal observation study.

The follow-up times for the patients were recorded until 2007 as follows: If no mammary carcinoma was reported in the data of our own practice and that of the Breast Cancer Center, the time from shoulder irradiation to 2007 was counted. For patients who were older than sixty years of age at the time of shoulder irradiation, the follow-up time was limited to the average remaining lifespan to be expected. Since starting from 2007 it was no longer certain that all cases of breast cancer in the district were being reported to us, the follow-up times were only extended beyond 2007 if the patients came in for a mammography examination with negative results or if they were examined in the clinic of Konstanz without any mammary findings. Patient without any information in our archive since radiotherapy of their shoulder were contacted personally, if they were still alive. Also relatives and family members were contacted when possible.

The estimation of the spontaneous incidence of mammary carcinoma for our collective was done as follows: For each patient, the average mammary carcinoma incidence for the life years from shoulder irradiation to the end of the observation period was determined from the tables of the “Society of Epidemiological Cancer Registries in Germany”^32^. This number was multiplied by the duration of observation, and finally the incidence values of all subjects were added. The 95% confidence interval (CI) can be derived from the fluctuating yearly incidence rates. The 100.000 inhabitants of our region create a yearly rate of breast cancer diseases of 112 cases in average^32^ with a variance of 112 as well (Poisson-statistics). The 95% CI results in the double standard deviation as 2*SQRT(112) = 21.2 or 19% from the average. This CI of the breast cancer diseases rate propagates in the determination of the predicted incidence interval, which is only a sum of incidence rates.

### Radiation technique

All the women were irradiated with a cobalt-60 device from Siemens, (Gammatron R). The dose rate was approximately 1.2 Gy/min at a distance of 50 cm; gamma quantum energies were 1.17 and 1.33 MeV.

### Radiation plan

A 10 × 10 cm^2^ or 12 × 12 cm^2^ a-p fixed field with focus-skin distance of 60 cm, collimator angle 30 degrees and sometimes an absorber block (over breast) was used. The median cumulative energy dose at the shoulder surface was 6 Gy, the single dose 1 Gy once a week.

### Dose estimation of the mammary glands

Generally treatment planning systems (TPS) show great uncertainties and deviations from the real dose out of the used field. With increasing distances from the field margin the uncertainty increases. This is the situation of the ipsilateral and contralateral mammary glands in the geometric relation to the irradiated shoulder. Present day treatment planning systems for a Co-60 device with the special geometry used 30 years ago for the treatment of the patients represented in our study are not available any more.

**Figure 1:**
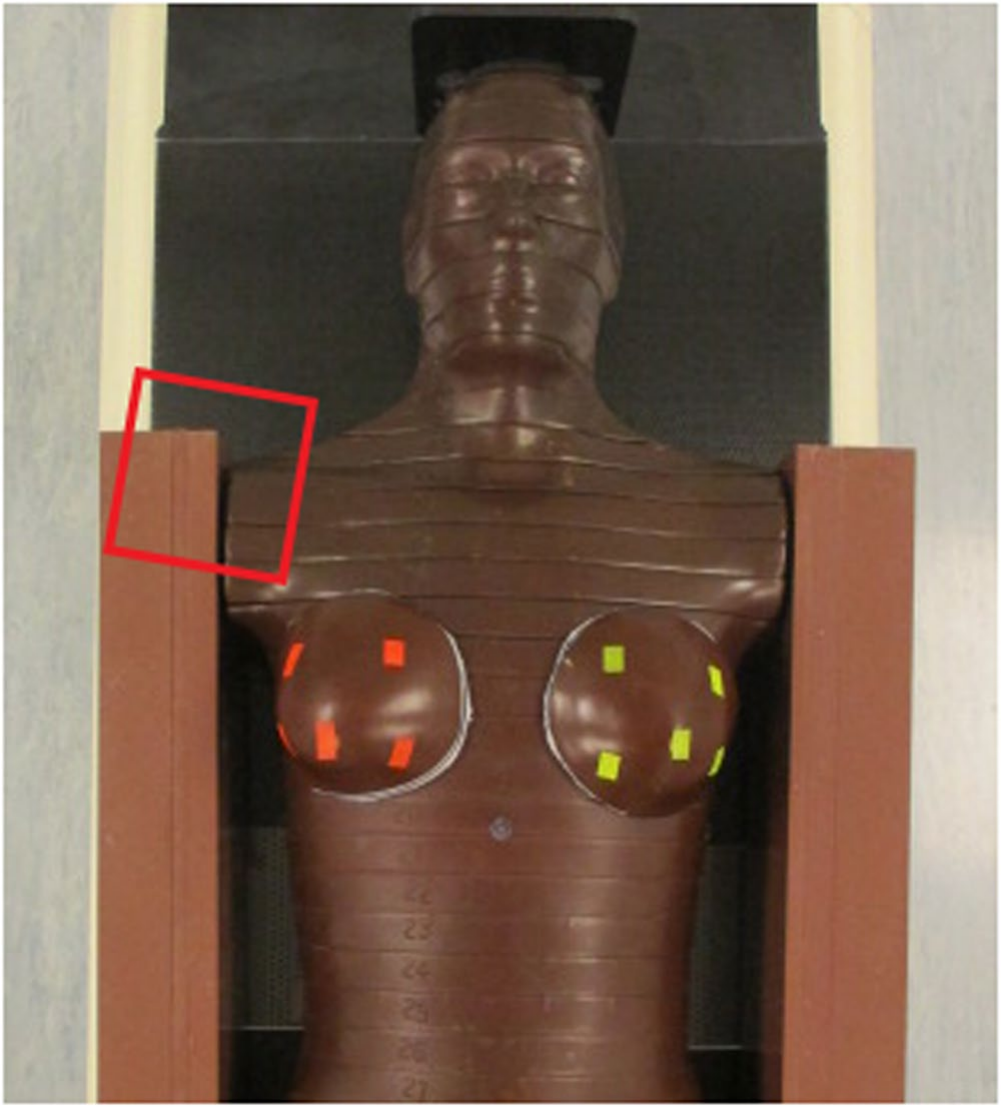
Female Alderson phantom with water equivalent slabs (thickness 8 cm) on each side mimicking the arms. TLD crystals were positioned on each breast as follows: two TLDs in each quadrant (one at the base and one at the surface), one in a borehole in the center of the breast and one at the papilla of the breast. The red (ipsilateral) and yellow (contralateral) marks show the locations of the TLDs at the surface of the breasts in relation to the Co-60 treatment field (12 × 12 cm) of the right shoulder. Between the torso and the breasts white thermoplastic material with a thickness of 6 mm was added, where the TLDs at the base of the breast were inserted.

To make allowance to these circumstances we used an anthropomorphic standard phantom (Alderson phantom, RSD Phantoms, CA, USA) in a female version; that means with mammary glands of 400 ml volume, each. To imitate the arms we added water equivalent slabs (thickness 8 cm, depth 15 cm, length 55 cm) at the shoulders. In this way we ensured to have full scattering. Thermoluminescent dosimeter (TLD) - crystals (LiF-100) packed in a PMMA (polymethylmethacrylate) tube (2.5 cm × 0.6 cm) were positioned on each breast as follows: two TLDs in each quadrant (one at the base and one at the surface), one in a borehole in the center of the breast and one at the papilla of the breast, see Fig. 1. The gamma ray irradiation of the shoulder was performed with a Cobalt-60 device (Gammabeam, Best Theratronics, Canada) from PTW (Physikalisch-Technische Werkstätten) in Freiburg, Germany. It has the same geometry as the one, which was used for patient treatment 30 years ago. The evaluation of the TLD elements was performed by PTW, a certified TLD laboratory, too.

### Statistics

The carried out statistics were performed in Sigma.Plot.10.0®.

The datasets generated during and/or analyzed during the current study are available from the corresponding author on reasonable request.

## Results

**Figure 2:**
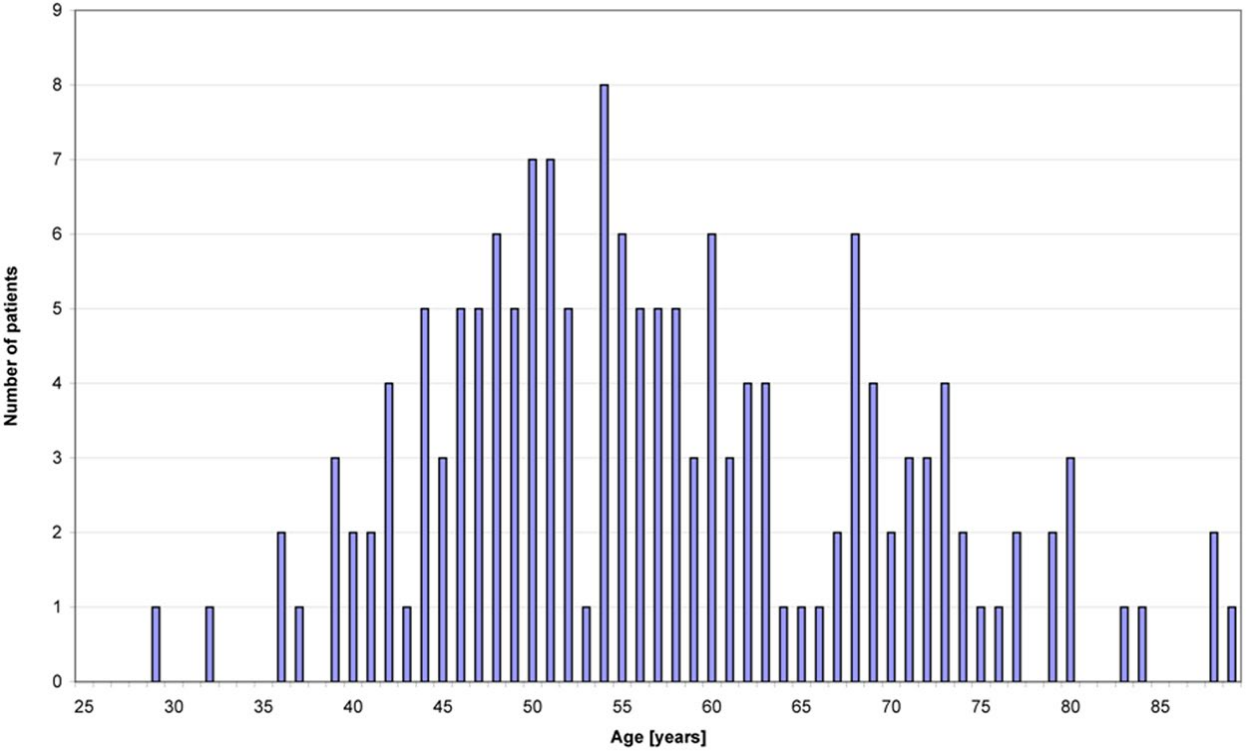
Patients’ age at irradiation of the shoulder.

**Figure 3:**
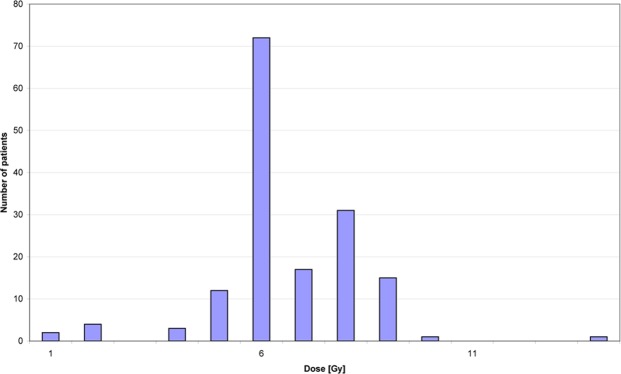
Distribution of the applied cumulative treatment dose within the patients’ cohort.

At the time of shoulder irradiation, the age of the 158 female patients was on average 57.1 years (range: 28–89 years) with a median of 55 years (Fig. 2). The distribution of the applied cumulative dose at the shoulder within the collective is illustrated in Fig. 3. The median cumulative dose was 6 Gy in the maximum of the fixed field, whereas the weekly single dose was 1 Gy. These values correspond to the median values of the total cohort.

**Figure 4:**
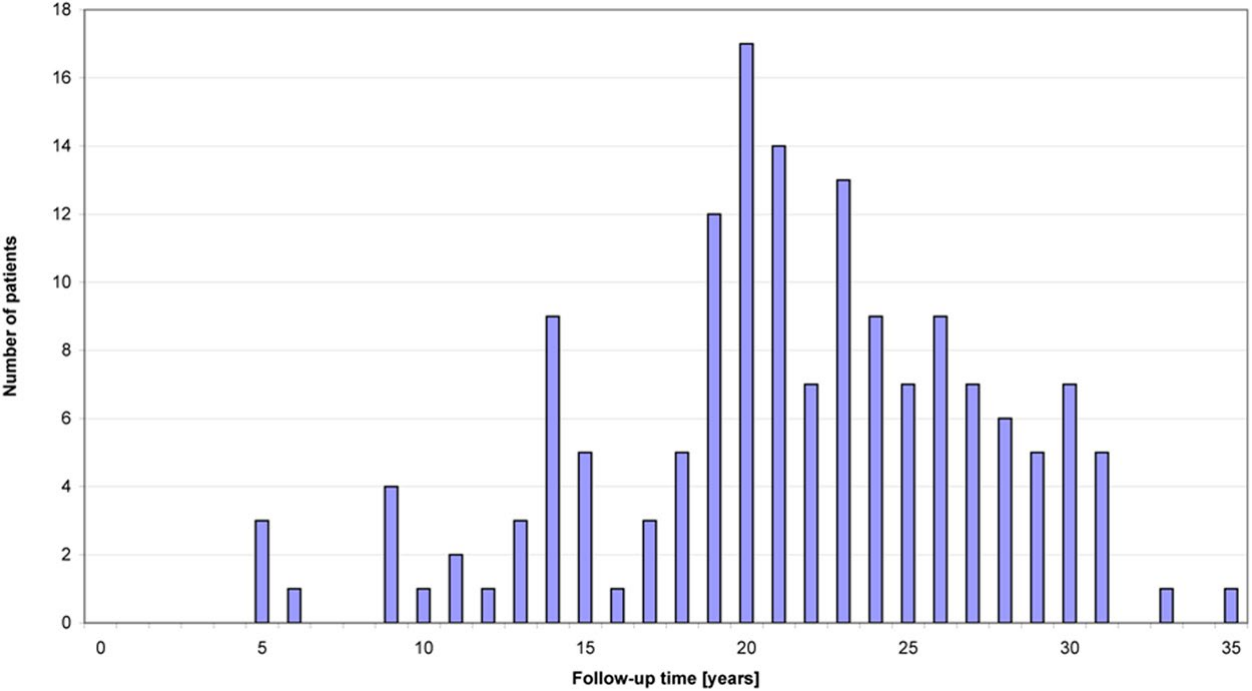
Follow-up times after irradiation of the shoulder.

**Figure 5:**
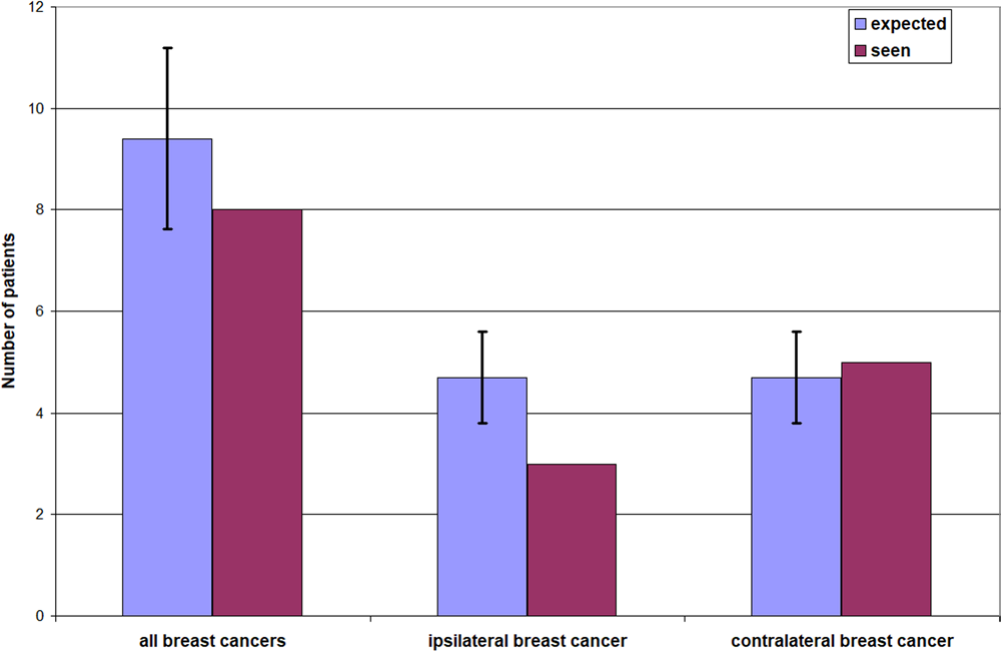
Seen and expected account of patients with breast cancer after low dose radiotherapy of the shoulder. Left pair of columns: total number of carcinomas, middle pair of columns: carcinomas ipsilateral to the shoulder irradiation, right: carcinomas contralateral to the shoulder irradiation. Bars show 95%CI.

The follow-up times after low dose radiation of the shoulder were 21.3 years on average and 21 years in the median (Fig. 4). Evaluation of the 158 female patients showed eight mammary carcinomas in seven patients (4.4%), whereas one patient showed bilateral carcinomas. Diagnosis of breast cancer was performed by biopsy and histological investigations. In one case only information of tumor suspect mammography (BI-RADS-5) was available. Table 1 summarizes various characteristics of the mammary carcinoma patients. According to the incidence statistics^32^, 9.4 +/− 1.8 (95%CI) cases (5.9%) would be expected. Therefore the actual incidence of breast cancer was not in excess of the expected incidence. (Fig. 5).

**Table 1:** Characteristics of patients with detected breast cancer.

Age at shoulder irradiation	side and applied dose	breast cancer years after shoulder RT	localisation of breast cancer	tumor formula	ipsi-/contralateral
Patient 1 59 years	right + left 6 Gy	20 years	left, upper, inner quadrant	pT1c N0 M0 hormone rec. positive	ipsilateral
Patient 2 55 years	right 6 Gy	15 years	right, upper, outer quadrant	pT1b N0 M0 hormone rec. positive, G1	ipsilateral
Patient 3 49 years	left 8 Gy	32 years	right, upper, inner quadrant	pT1c N0 M0	contralateral
Patient 4 56 years	right 2 Gy	28 years	right + left	>/=pT1	ipsi- + contralateral
Patient 5 60 years	left 6 Gy	11 years	right upper, inner quadrant	pT1b N0 M0	contralateral
Patient 6 51 years	left 9 Gy	35 years	right upper, outer quadrant	>/=pT1	contralateral
Patient 7 50 years	right 5 Gy	17 years	left upper, outer quadrant	pT1c N0 M0	contralateral

The time span between shoulder irradiation and diagnosis of mammary carcinoma was between 11 and 35 years, the median time span was 20 years. Localization of the mammary carcinoma was contralateral to the shoulder irradiation five times and ipsilateral three times. Here the carcinoma of patient 1, which occurred after simultaneous irradiation of both shoulders, was evaluated as ipsilateral due to the high scatter dose. All carcinomas found were in the upper quadrants, three outer and three inner (Table 1).

Alderson phantom measurements using TLDs for a typical irradiation of the right shoulder with 6 Gy surface dose, show the following results for the ispilateral and contralateral mammary gland. For the ipsilateral breast the measured doses ranged from max. 41 mGy (surface of the upper outer quadrant) to 14 mGy (lower inner quadrant). In the center the dose-value was 20mGy. The mean dose of the ispilateral breast was 25 mGy (SD +/− 11 mGy). For the contralateral breast the doses ranged from 16 mGy to 6 mGy. In the center the dose value was 10 mGy. The mean dose of the contralateral breast was 11 mGy (SD +/− 4 mGy). The average dose for both breasts was 18 mGy (SD +/− 11 mGy).

## Discussion

In this long-term study the breast cancer occurrence after low dose radiotherapy of non-malignant disorders of the shoulder was investigated. An induction of additional breast cancer after radiotherapy of non-malignant disorders of the shoulder was not detected in the analyzed cohort. In regard to the irradiated shoulder neither the ipsilateral nor the contralateral breasts showed increased rates of breast cancer.

A central issue of radiation therapy is the induction of malignancies, which is always possible with a statistical probability. This induction depends on many factors such as the applied dose and dose rate, the treated volume, the sensitivity of the irradiated organ^19^ and the age of the individual^33^. As recommended in ICRP 2008^19^ and in the publication of Trott and Kamprad^20^, malignancy risk calculations according to the model of “effective dose” should be regarded critically and compared with clinical data.

For this purpose, we retrospectively investigated the incidence of breast cancer in a female collective that had been subjected to non-malignant irradiation of the shoulder and made a comparison with the estimated spontaneous incidence of mammary carcinoma for this collective. The estimated scattered radiation doses applied in the ipsilateral mammary gland averaged 25 mGy with a maximum of 41 mGy in the upper outer quadrant. The contralateral mammary gland received a mean dose of 11 mGy. Those values are partly as high as usual doses in radiological diagnostic procedures, but clearly lower than those in tumor radiotherapy.

In case of low dose radiotherapy of non-malignant disorders it is reported, that treatment of ankylosing spondylitis with great treatment-fields (parts or whole bone spine) and doses between 4–6 Gy increases the risk of leukaemia and cancer. In the reported cohort patients were relative young (45 years) and the estimated mean absorbed red bone marrow dose was 3.38Gy^25–27,34^. In contrast low dose irradiation of the shoulder causing a mean absorbed red bone narrow dose of 0.11 Gy did not increase rates of haematological malignancies^28,29^.

To our knowledge clinical data about induction of breast cancer after radiotherapy of non-malignant disorders of the shoulder haven’t been published yet. In the cohort of 158 subjects that we investigated, we found seven cases of mammary carcinoma. In order to decide whether these carcinomas are spontaneous or radiation-induced, we estimated the spontaneous incidence of mammary carcinoma for our cohort as follows: For each patient, the average mammary carcinoma incidence for the life years from shoulder irradiation to the end of the observation period was determined from the tables of the “Society of Epidemiological Cancer Registries in Germany”^32^. This number was multiplied by the duration of observation, and finally the incidence values of all subjects were added. The result is: For the 158 patients evaluated, 9.4 +/− 1.8 (95%CI) mammary cancer cases are to be expected in the observation time, which comes close to our observed incidence of seven women with eight mammary carcinomas. Therefore, the cancer cases found are to be evaluated as spontaneous. Figure 5 shows these results graphically.

The localization of the mammary carcinomas found, which all occurred in the two upper quadrants of the mammary glands, also speaks for this. According to the literature^35,36^, the frequency of mammary carcinomas in the two upper quadrants is up to 70% of the total number.

A further argument against radiation induction is the lack of dose dependency of the cancer cases observed. The breast located ipsilaterally to the shoulder radiation, which was clearly subjected to more radiation, does not show more carcinoma cases than the contralaterally located breast. The fact that the contralateral side shows even more carcinomas is random (see Fig. 5).

For the latency period between radiation exposure and the occurrence of a mammary carcinoma, the following numbers are known: In atom bomb victims from 1945, mammary carcinomas did not appear more frequently until more than 10 years later^37^. The same is shown by studies with medical diagnostic radiation exposure^38^. A meta-analysis of M. Hodgkin’s collectives shows a latency period of seven to 30 years, median 18 years^39,40^. Our follow-up times of 11 to 35 years, median 20 years, were long enough to show a radiation-induced effect.

It is known from the literature^33,41^ that the dose-effect curve of radiation-induced mammary carcinoma can be described with a linear-quadratic model. Here, the carcinogenic risk increases linearly at organ doses of less than 10 Gy and shows saturation from approximately 20 Gy. The doses in our collective of less than 1 Gy were clearly in the linear part of the dose-effect curve.

Calculation of the induced frequency of malignancies in the breast, in the “effective dose” model for our collective of 158 patients, results in a value of approx. 0.02 persons. In detail: detriment-adjusted cancer risk = 0.05 Sv^−1^ × 158 × 0.12 × 0.018 Sv ≈ 0.02^19^. Here, the nominal probability coefficient for detriment-adjusted cancer risk is 0.05 Sv^−1^. The numbers provided by the ICRP of w_T_ = 0.12 for the breast and DDREF (dose and dose-rate effectiveness factor) = 1 for single doses in the mammary glands less than 0.2 Gy were used. The equivalent dose H = 0.018 Sv corresponds to the energy dose E = 0.018 Gy. For the worst case we assumed the measured maximum dose (0.041 Gy) in the upper outer quadrant of the ipsilateral breast, which is the area of the breast with the highest incidence of breast cancer in common^35,36^. When calculating with this dose value, we expect 0.04 persons with radiation induced breast cancer for our collective.

The measured doses in the ipsilateral and contralateral breast represent the dose in the case of an anthropomorphic standard anatomy. Dose uncertainties have to be considered regarding interindividual variations and differences in the female anatomy.

The “effective dose” model thus does not predict any recognizably increased frequency of malignancies for our collective. This very conservatively calculated number is confirmed by our result that we did not detect any increased frequency of mammary carcinoma in our collective. Our clinical data show that the “effective dose” model does not underestimate the risk of secondary malignancy after low dose radiotherapy. To prove if the “effective dose” model does not overestimate this risk, epidemiological studies with greater patients’ numbers are necessary. Also prospective data acquisition of patients with radiotherapy of non-malignant disorders over an extended period would be desirable but just as difficult.

The suggested life time risk of cancer induction is approximately 0.2% after low dose irradiation. The patients’ age is also an important factor for irradiation dependent cancer induction. It is also reported, that the carcinogenic risk of radiotherapy of benign diseases is decreased when treated patients are older than 40 years^1,6,30,31^. The median age of the patients in this collective was 55 years.

Preston *et al*. discussed the age-dependency of breast cancer induction^42^. They summarized data from several cohorts of different ages. We calculated the additional expected number of irradiation induced breast cancers for our collective by using an EAR (Excess absolute risk) of 9.9 (10^4^ wy Gy)^−1^ (from Table 12, Preston *et al*.)^42^ as follows: 9.9 (10^4^ wy Gy)^−1^ × 0.041 (Gy) × 0.337 (10^4^ wy) × 0.216 (for a three decades elder cohort)^42^ = 0.03.

In the daily routine indication and execution of low dose radiotherapy of non-malignant disorders of the shoulder has to be done accurately. Patients’ age, size of the planning target volume and also of the applied dose should to be considered. Because of uncertainties of scattered radiation in the breast by interindividual variations in the female anatomy, maximal protection of the mammary glands is generally required in radiotherapy planning.

## Conclusion

Within the bounds of a retrospective analysis there is no evidence of an induction of additional breast cancer by radiation of non-malignant disorders of the shoulder in this cohort. Neither an excess of mammary carcinoma nor dose dependency of possible carcinoma induction could be shown.
